# Supplementary material for: Macrostructural and Microstructural White Matter Alterations Are Associated with Apathy across the Clinical Alzheimer’s Disease Spectrum
Source: Brain Sci. 2022 Oct 13;12(10):1383. doi: 10.3390/brainsci12101383 (PMC9599811; doi:10.3390/brainsci12101383)
Supplement: Supplementary file 1 [file brainsci-12-01383-s001.zip › brainsci-1947572-supplementary.pdf]

Table S1. Differences in the prevalence of individual neuropsychiatric symptoms (other than apathy) between patient groups (*Chi-square* test). All values are frequencies (proportions).

Symptom	AP-PT (*n* = 61)	NA-PT (*n* = 61)	F	*p*
Delusions	6 (9.8%)	1 (1.6%)	-[a]	0.114
Hallucinations	1 (1.6%)	0 (0.0 %)	-[a]	1.000
Agitation	27 (44.3%)	12 (19.7%)	8.48	0.004
Depression	37 (60.6%)	11 (18.0%)	23.22	<0.001
Anxiety	21 (34.4%)	9 (14.7%)	6.36	0.012
Euphoria	6 (9.8%)	1 (1.6%)	-[a]	0.114
Disinhibition	16 (26.2%)	1 (1.6%)	15.38	<0.001
Irritability	37 (60.6%)	10 (16.4%)	25.23	<0.001
Aberrant motor behaviours	10 (16.4%)	0 (0.0 %)	10.89	<0.001
Night-time behaviours	23 (37.7%)	15 (24.6%)	2.45	0.118
Appetite	16 (26.2%)	6 (9.8%)	5.54	0.019

[a] *Fisher's Exact* Test

Table S2. Differences in the prevalence of health issues in the medical history of participant groups (*Chi-square* test). All values are frequencies (proportions).

Comorbid health conditions	AP-PT (*n* = 61)	NA-PT (*n* = 61)	CU (*n* = 61)	F	*p*
Psychiatric	**34 (55.7%)***	**20 (32.8%)**	12 (19.7%)	17.63	<0.001
Neurologic (other than AD)	12 (19.7%)	13 (21.3%)	13 (21.3%)	0.07	0.967
Head, eyes, nose and throat	34 (55.7%)	42 (68.9%)	37 (60.7%)	2.27	0.322
Cardiovascular	46 (75.4%)	42 (68.9%)	44 (72.1%)	0.65	0.722
Respiratory	17 (27.9%)	14 (23.0%)	7 (11.5%)	5.24	0.073
Hepatic	2 (3.3%)	2 (3.3%)	1 (1.6%)	0.41	0.814
Dermatologic	22 (36.1%)	16 (26.2%)	18 (29.5%)	1.44	0.487
Musculoskeletal	40 (65.6%)	38 (62.3%)	42 (68.9%)	0.58	0.748
Endocrine-Metabolic	32 (52.5%)	26 (42.6%)	34 (55.7%)	2.27	0.321
Gastrointestinal	22 (36.1%)	30 49.2%)	28 (45.9%)	2.31	0.315
Hematopoietic-Lymphatic	4 (6.6%)	5 (8.2%)	7 (11.5%)	0.96	0.619
Renal-Genitourinary	18 (29.5%)	25 (41.5%)	20 (32.8%)	1.89	0.389
Allergies-Drug sensitivity	24 (39.3%)	30 49.2%)	26 (42.6%)	1.24	0.537
Alcohol abuse	2 (3.3%)	0 (0.0%)	1 (1.6%)	2.03	0.362
Drug abuse	0 (0.0%)	0 (0.0%)	0 (0.0%)	0.00	1.000
Smoking	25 (41.5%)	26 (42.6%)	28 (45.9%)	0.31	0.856
Malignancy	20 (32.8%)	14 (23.0%)	18 (29.5%)	1.50	0.471

Major Surgical procedures	46 (75.4%)	43 (70.5%)	42 (68.9%)	0.70	0.705

AD: Alzheimer's disease

* Patient group significantly different from the CU group in pairwise *post hoc* comparisons
In bold, significant difference between patient groups in pairwise *post hoc* comparisons

Table S3. Differences in the prevalence of mental health problems in the medical history of participant groups (*Chi-square* test). All values are frequencies (proportions).

**Symptoms**	**AP-PT (*n* = 61)**	**NA-PT (*n* = 61)**	**CU (*n* = 61)**	**F**	***p***
Depression	28 (45.9%)*	18 (29.5%)*	8 (13.1%)	15.76	<0.001
Insomnia	4 (6.6%)	4 (6.6%)	1 (1.6%)	2.51[a]	0.285
Nervous breakdown	1 (1.6%)	0 (0.0%)	0 (0.0%)	2.21[a]	0.332
Anxiety	5 (8.2%)	3 (4.9%)	2 (3.3%)	1.46[a]	0.482
Irritability	1 (1.6%)	1 (1.6%)	0 (0.0%)	1.63[a]	0.442
Seasonal affective disorder	0 (0.0%)	0 (0.0%)	1 (1.6%)	2.21	0.332

[a] Likelihood ratio

* Patient groups significantly different from the CU group in pairwise *post hoc* comparisons

Table S4. Differences in the prevalence of medications with a potential impact on apathy across participant groups (*Chi-square* test). All values are frequencies (proportions).

**Treatment**	**AP-PT (*n* = 61)**	**NA-PT (*n* = 61)**	**CU (*n* = 61)**	**F**	***p***
AChEI	33 (54.1%)*	25 (41.0%)*	0 (0.0 %)	44.88	<0.001
Memantine	**17 (27.9%)***	**8 (13.1%)***	0 (0.0 %)	20.11	<0.001
Antidepressants	**23 (37.7%)***	**12 (19.7%)***	3 (4.8%)	19.99	<0.001
Other behavioural medications	2 (3.2%)	0 (0.0 %)	1 (1.6%)	2.81[a]	0.246

AChEI: Acetylcholinesterase inhibitors

[a] Likelihood ratio

* Patient groups significantly different from the CU group in pairwise *post hoc* comparisons
In bold, significant difference between patient groups in pairwise *post hoc* comparisons

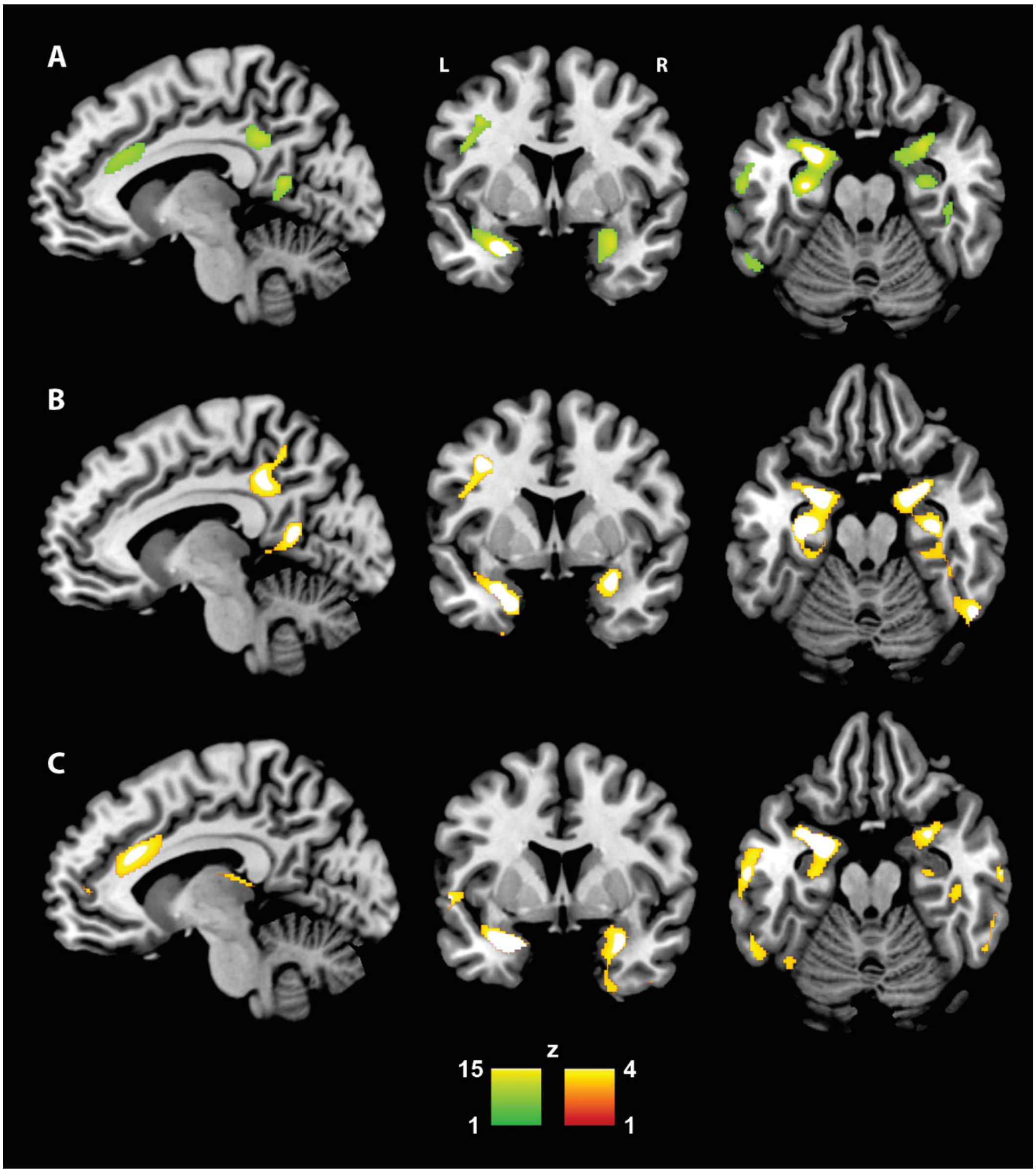

Figure S1. Significant regional GM volume differences (FWE-corrected at cluster level): A. *ANCOVA*; B. NA-PT < CU group; C. AP-PT < CU group.

Abbreviation list.

Aβ: Amyloid beta, ACC: Anterior cingulate cortex, AChEI: Acetylcholinesterase inhibitors, AD: Alzheimer's disease, ADNI: Alzheimer's Disease Neuroimaging Initiative, ANCOVA: Analysis of covariance, ANOVA: Analysis of variance, AP-PT: Apathetic patients, ATR: Anterior thalamic radiations, AVLT: Auditory Verbal Learning Test, AxD: Axial diffusivity, BNT: Boston Naming Test, CDR: Clinical Dementia Rating, CDT: Clock Drawing Test, CFT-A: Category Fluency Test - animals, CSF: Cerebrospinal fluid, CU: Cognitively unimpaired, DR: Delayed recall, DTI: Diffusion tensor imaging, FA: Fractional anisotropy, FLAIR: Fluid-attenuated inversion recovery, FSL: Functional magnetic resonance imaging of the brain Software Library, FWE: Family-wise error, GM: Gray matter, GMV: Gray matter volume, ICBM: International Consortium for Brain Mapping, IFL: Inferior longitudinal fasciculus, IFOF: Inferior fronto-occipital fasciculus, IR: Immediate recall, LMT: Logical Memory Test, LST: Lesion Segmentation Toolbox, MCI: Mild cognitive impairment, MD: Mean diffusivity, MMSE: Mini Mental State Examination, MNI: Montreal Neurological Institute, NA-PT: Non-apathetic patients, NPI-Q: Neuropsychiatric Inventory Questionnaire, OFC: Orbitofrontal cortex, p-tau: Phosphorylated tau, PET: Positron emission tomography, PFC: Prefrontal cortex, RD: Radial diffusivity, SLF: Superior longitudinal fasciculus, SPM: Statistical Parametric Mapping, TBSS: Tract-based spatial statistics, TFCE: Threshold-free cluster enhanced, TIV: Total intracranial volume, TMT – A/B: Trail Making Test – part A/part B, VBM: Voxel-based morphometry, WM: White matter, WMH: White matter hyperintensity, WMHV: White matter hyperintensity volume, WMV: White matter volume
